# Generalized keratosis pilaris rubra mimicking erythromelanosis follicularis faciei et colli: a case report

**DOI:** 10.1097/MS9.0000000000004857

**Published:** 2026-03-31

**Authors:** Sandesh Shah, Joshana Shrestha, Mohan Bhusal, Deepika Neupane, Radhika Maharjan

**Affiliations:** a Department of Dermatology, Nepal Medical College and Teaching Hospital, Kathmandu, Nepal; b Department of Prosthodontics, B.P Koirala Institute of Health Sciences, Dharan, Nepal

**Keywords:** erythromelanosis follicularis faciei et colli, keratosis pilaris, keratosis pilaris rubra

## Abstract

**Introduction and Importance:**

Keratosis pilaris rubra (KPR) is a benign follicular disorder characterized by erythematous papules and perifollicular inflammation, typically affecting adolescents and young adults. The generalized form is rare and diagnostically challenging due to overlap with erythromelanosis follicularis faciei et colli (EFFC).

**Case Presentation:**

A 19-year-old obese Buddhist male presented with longstanding erythema and rough, bumpy papules over the cheeks, neck, trunk, and all four extremities, associated with burning sensations. Dermoscopy showed follicular keratotic plugs, perifollicular erythema, a diffuse reddish background, and multiple linear vessels. Histopathology of a cheek biopsy demonstrated follicular infundibular plugging with perifollicular lymphocytic infiltrate, supporting KPR. Topical tretinoin 0.05%, mometasone 0.1% cream, urea-based moisturizer, and sunscreen led to mild improvement in texture, with persistent erythema.

**Clinical Discussion:**

Generalized KPR is uncommon and can be mistaken for EFFC owing to overlapping clinical and dermoscopic features. Dermoscopy (follicular plugs, perifollicular erythema, linear vessels) and histology (infundibular plugging, perifollicular lymphocytes) help distinguish KPR, while EFFC typically shows pigmentary changes and dermoscopic peppering. Therapeutic options beyond topical agents include pulsed dye laser and topical sirolimus in selected cases.

**Conclusion:**

This case underscores the diagnostic complexity of generalized KPR and the importance of correlating clinical, dermoscopic, and histopathological findings. Conventional therapies provide limited benefit, and financial constraints may restrict access to advanced options like laser treatment.

## Introduction and importance

Keratosis pilaris rubra (KPR) is a chronic, benign skin condition marked by follicular keratotic papules with persistent erythema and heightened inflammatory/vascular features, typically seen in adolescents and young adults^[^1,2^]^. Although often considered cosmetic, redness, burning, and extensive distribution can affect quality of life. Generalized KPR, involving the face, neck, trunk, and limbs, is rare and more challenging to diagnose and treat^[^3^]^. Histopathology shows follicular infundibular plugging with perifollicular lymphocytic infiltration, while dermoscopy reveals follicular plugs, perifollicular erythema, a diffuse reddish background, and linear/coiled vessels^[^1,4^]^. Differentiation from erythromelanosis follicularis faciei et colli (EFFC) – characterized by erythema, hyperpigmentation, and follicular papules – relies on identifying melanin-related pigmentation and dermoscopic peppering^[^5–7^]^. In line with best practice, we present this case following structured reporting principles, referencing the TITAN checklist^[^8^]^.

HIGHLIGHTS

- Generalized keratosis pilaris rubra (KPR) is a rare and underreported variant that can mimic other follicular disorders.
- Differentiation from erythromelanosis follicularis faciei et colli (EFFC) is challenging due to overlapping clinical and dermoscopic features.
- Dermoscopy and histopathology are critical for distinguishing generalized KPR from EFFC.
- Treatment response with conventional topical agents remains limited, highlighting the need for advanced therapies such as lasers or sirolimus.

## Case presentation

A 19-year-old obese Buddhist male presented to the dermatology outpatient department with persistent facial redness and rough, bumpy skin lesions present for several years, associated with burning sensations. There was no prior medical, family, or drug history, and no atopy in the patient or family.

**Figure 1.**
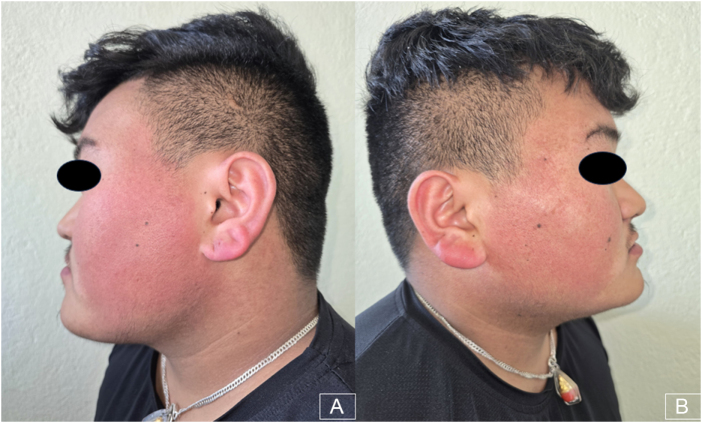
Multiple small, spiny, monomorphic follicular keratotic papules with confluent surrounding erythema over bilateral cheeks extending to the neck.

**Figure 2.**
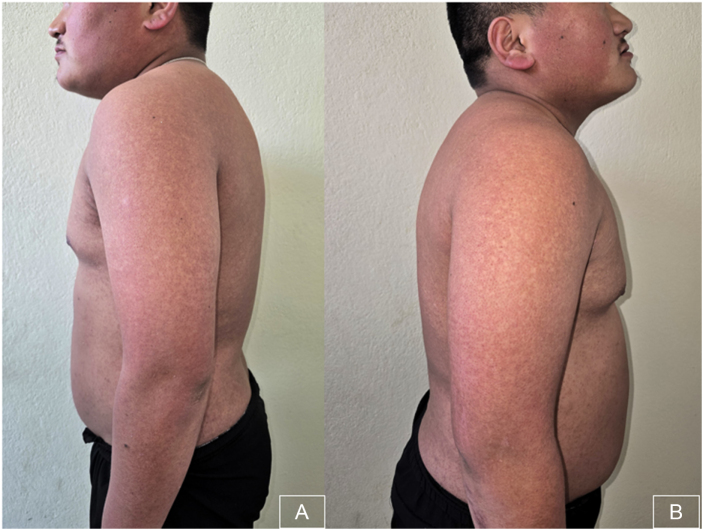
Multiple small, spiny, monomorphic follicular keratotic papules with confluent surrounding erythema over bilateral upper extremity and trunk.

**Figure 3.**
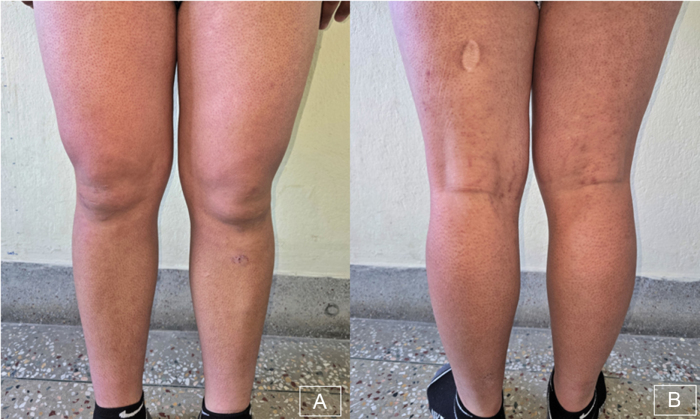
Multiple small, spiny, monomorphic follicular keratotic papules with confluent surrounding erythema over bilateral lower extremities.

Examination revealed multiple small, spiny, monomorphic follicular keratotic papules with confluent surrounding erythema over the bilateral cheeks extending to the lateral neck (Fig. 1). Similar lesions were present on the trunk and on both upper and lower extremities (Figs 2 and 3). Lesions were non-tender and non-pruritic, with no signs of infection, scarring, or alopecia.

**Figure 4.**
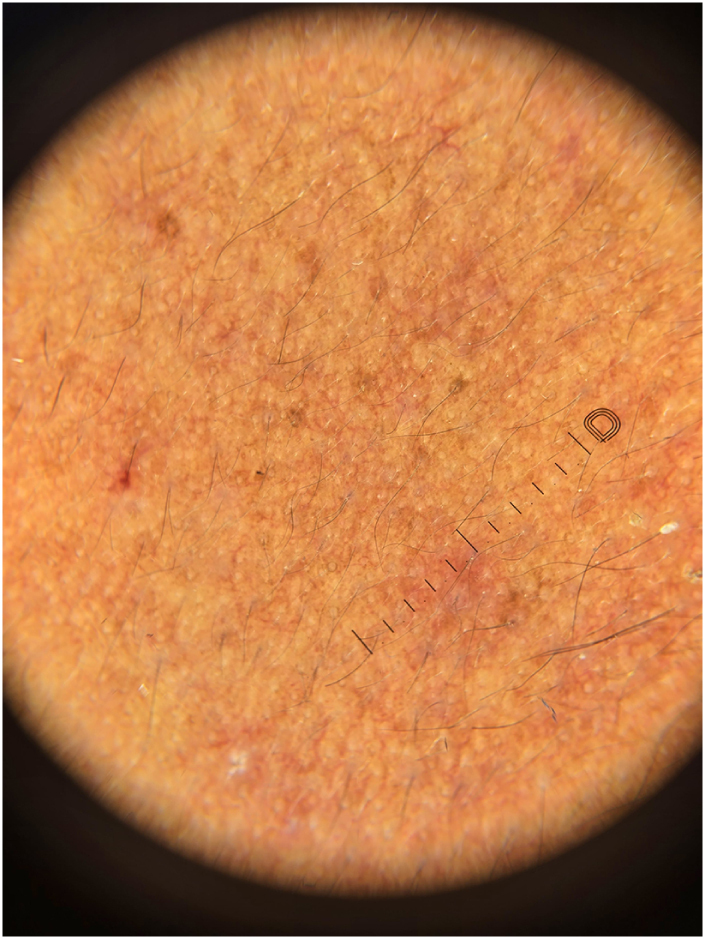
Follicular keratotic plugs, perifollicular erythema, and a diffuse reddish background, along with the presence of multiple linear vessels.

**Figure 5.**
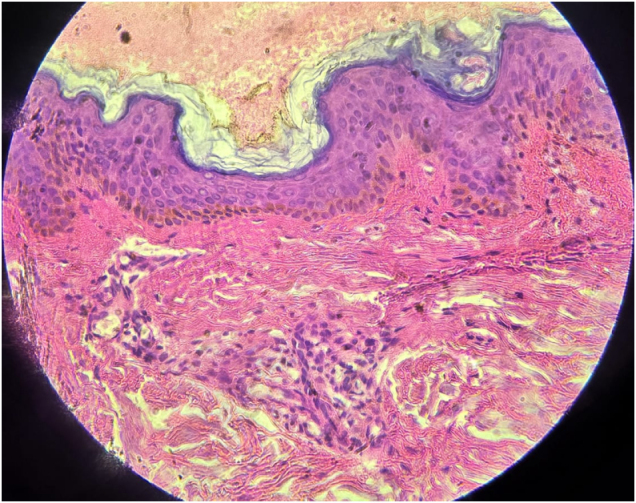
Hematoxylin and eosin stain at 40× showing hyperkeratosis, follicular infundibular plugging along with a mild perifollicular lymphocytic infiltrate.

Dermoscopy of the affected skin showed follicular keratotic plugs, perifollicular erythema, and a diffuse reddish background with multiple linear vessels (Fig. 4). Laboratory tests (CBC, LFTs, RFTs) were within normal limits, and ANA was negative. A punch biopsy from the lateral cheek showed hyperkeratosis, follicular infundibular plugging, and mild perifollicular lymphocytic infiltrate (Fig. 5), consistent with keratosis pilaris.

The patient was treated with topical tretinoin 0.05%, mometasone 0.1% cream on erythematous areas, urea-based moisturizer, and broad-spectrum sunscreen. At 4 weeks, there was mild improvement in texture, with persistent erythema. The patient was counseled regarding laser therapy; however, he deferred the treatment owing to financial constraints.

## Clinical discussion

KPR mainly involves the face, lateral neck, and upper arms and is characterized by erythematous follicular papules with perifollicular inflammation. Generalized KPR, which affects the trunk and lower extremities as seen in our patient, is rare and sparsely documented^[^1,2^]^. Dermoscopy typically reveals follicular plugs, perifollicular erythema, and vascular structures (e.g., linear vessels), consistent with our observations^[^4^]^. Histologically, infundibular plugging, hyperkeratosis, and mild perifollicular lymphocytic infiltrate are expected and were observed in our biopsy^[^2,3^]^. Distinguishing KPR from EFFC is challenging due to overlapping features. EFFC generally presents with erythema, follicular papules, and increased pigmentation, especially in darker skin types, and may coexist with keratosis pilaris^[^6^]^. Dermoscopically, EFFC shows whitish follicular keratotic plugs over a reddish-brown background with gray-blue granules (peppering) and white scales, correlating with pigmentary incontinence and dermal melanophages histologically^[^6,7^]^. The absence of dermoscopic peppering and the prominence of perifollicular erythema support a diagnosis of KPR in our case. Management of KPR is mainly symptomatic. Topical keratolytics, retinoids, and corticosteroids often provide limited improvement, especially for persistent erythema^[^1,3^]^. Evidence supports using 595-nm pulsed dye laser for erythema reduction and topical sirolimus in select cases^[^1,3^]^. Access and cost may limit these advanced treatments.

## Conclusion

This case illustrates the diagnostic challenges of generalized KPR, a rare entity often mistaken for EFFC due to overlapping features. Accurate diagnosis requires careful integration of clinical, dermoscopic, and histopathological findings. Conventional topical therapies provide limited benefit, particularly for persistent erythema, and resource limitations may hinder access to advanced modalities such as laser therapy. Greater awareness of this uncommon presentation can improve recognition, reduce misdiagnosis, and guide more effective patient counseling and management.

## Data Availability

Not applicable.
